# Age of the Association between *Helicobacter pylori* and Man

**DOI:** 10.1371/journal.ppat.1002693

**Published:** 2012-05-10

**Authors:** Yoshan Moodley, Bodo Linz, Robert P. Bond, Martin Nieuwoudt, Himla Soodyall, Carina M. Schlebusch, Steffi Bernhöft, James Hale, Sebastian Suerbaum, Lawrence Mugisha, Schalk W. van der Merwe, Mark Achtman

**Affiliations:** 1 Max-Planck-Institut für Infektionsbiologie, Department of Molecular Biology, Berlin, Germany; 2 Konrad Lorenz Institute for Ethology, Department of Integrative Biology and Evolution, University of Veterinary Medicine Vienna, Vienna, Austria; 3 Department of Biochemistry and Molecular Biology, Pennsylvania State University, University Park, Pennsylvania, United States of America; 4 Hepatology and GI-Research Laboratory, Department of Immunology, University of Pretoria, Pretoria, South Africa; 5 Human Genomic Diversity and Disease Research Unit, Division of Human Genetics, School of Pathology, University of the Witwatersrand/National Health Laboratory Services, Johannesburg, South Africa; 6 Environmental Research Institute and Department of Microbiology, University College Cork, Cork, Ireland; 7 Institute of Medical Microbiology and Hospital Epidemiology, Hannover Medical School, Hannover, Germany; 8 Ngamba Island Chimpanzee Sanctuary, Entebbe, Uganda; Yale University, United States of America

## Abstract

When modern humans left Africa ca. 60,000 years ago (60 kya), they were already infected with *Helicobacter pylori*, and these bacteria have subsequently diversified in parallel with their human hosts. But how long were humans infected by *H. pylori* prior to the out-of-Africa event? Did this co-evolution predate the emergence of modern humans, spanning the species divide? To answer these questions, we investigated the diversity of *H. pylori* in Africa, where both humans and *H. pylori* originated. Three distinct *H. pylori* populations are native to Africa: hpNEAfrica in Afro-Asiatic and Nilo-Saharan speakers, hpAfrica1 in Niger-Congo speakers and hpAfrica2 in South Africa. Rather than representing a sustained co-evolution over millions of years, we find that the coalescent for all *H. pylori* plus its closest relative *H. acinonychis* dates to 88–116 kya. At that time the phylogeny split into two primary super-lineages, one of which is associated with the former hunter-gatherers in southern Africa known as the San. *H. acinonychis*, which infects large felines, resulted from a later host jump from the San, 43–56 kya. These dating estimates, together with striking phylogenetic and quantitative human-bacterial similarities show that *H. pylori* is approximately as old as are anatomically modern humans. They also suggest that *H. pylori* may have been acquired *via* a single host jump from an unknown, non-human host. We also find evidence for a second Out of Africa migration in the last 52,000 years, because hpEurope is a hybrid population between hpAsia2 and hpNEAfrica, the latter of which arose in northeast Africa 36–52 kya, after the Out of Africa migrations around 60 kya.

## Author Summary

We previously showed that the population history of *H. pylori* may be used as a marker for human migrations, including the demonstration that humans carried *H. pylori* out of Africa 60,000 years ago during their recent global expansions. But how long were humans infected by *H. pylori* prior to the out-of-Africa event? Here we showed that chimpanzees in Central-East Africa do not possess *Helicobacter*-like bacteria, as would have been expected for pathogen-host co-evolution over millions of years. Using *H. pylori* gene sequences isolated from San, a group of click-speaking hunter-gatherers, and numerous other sources, we calculated that humans have been infected with *H. pylori* for at least 88,000–116,000 years. Phylogenetic comparisons showed similar evolutionary histories for human and *H. pylori* lineages and suggest that this association stemmed from a single host jump. We showed that hpAfrica2, the most divergent *H. pylori* population, arose in the San and that their progenitors were the source of *H. acinonychis* which was acquired by large felines approximately 50,000 years ago. Furthermore, our data provided clear evidence for a recent second exodus Out of Africa in the last 52,000 years which was essential for the formation of the hybrid population that currently infects Europeans.

## Introduction

**Figure 1:**
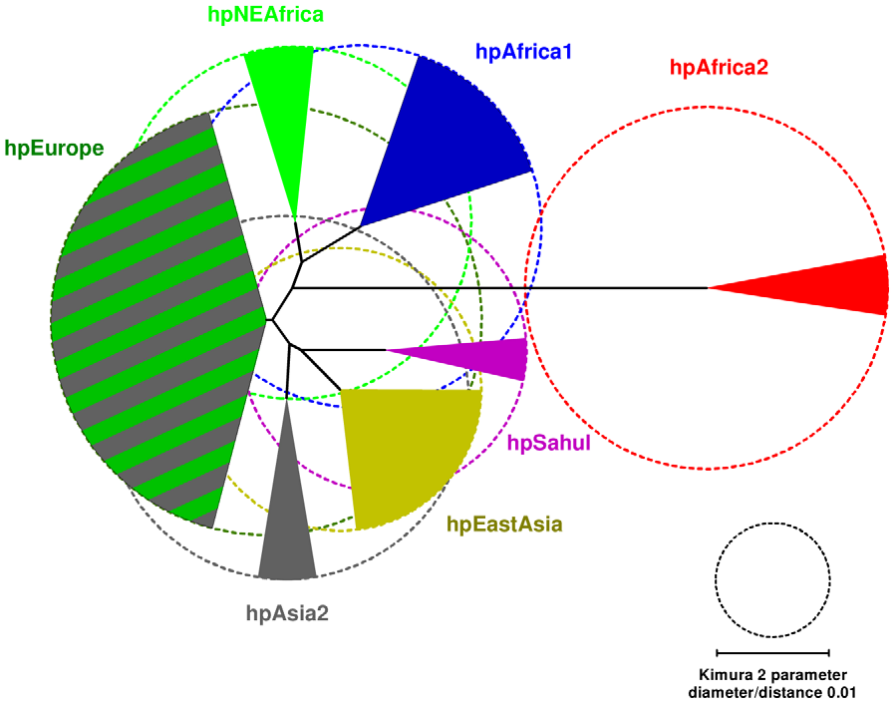
Neighbor-joining population tree of extant populations of *H. pylori*. Circle diameters are proportional to the within-population genetic diversity (π). Angles of filled arcs are proportional to the number of isolates. Data are from [2], [6]–[8] and the figure is modified from Figure 1 in [6].

The Gram-negative bacterium *Helicobacter pylori* infects the stomachs of at least 50% of all humans, causing gastric inflammation in all infected individuals, gastric or duodenal ulcers in 10–15% and gastric carcinoma or lymphoma of the mucosa-associated lymphoid tissue in ∼1% [1]. *H. pylori* infection is predominantly transmitted within families [2], suggesting that transmission requires intimate contact. Familial transmission has resulted in strong phylogeographic signals within these bacteria [3] due to the frequent, local dispersion of single nucleotide polymorphisms by homologous recombination [4]. At the global level, *H. pylori* has been subdivided by population genetic tools such as Structure [5] into multiple, relatively distinct populations that are specific for large geographical areas: hpEurope, hpSahul, hpEastAsia, hpAsia2, hpNEAfrica, hpAfrica1 and hpAfrica2 (Figure 1) [6]–[8]. The partitioning of genetic variation in *H. pylori* was more discriminatory in determining the ancient sources of human migrants in northern India [9], Southeast Asia [10] and the Pacific [8] than were traditional human genetic measures, such as the hypervariable segment 1 of the mitochondrial DNA control region.

Phylogeographic patterns in *H. pylori* have been shown to reflect significant demographic events in human prehistory [6], [11]. *H. pylori* has accompanied anatomically modern humans since their migrations out of Africa some 60,000 years ago (60 kya), and mirrors the human pattern of increased genetic distance and decreased diversity with distance from Africa [7]. However, the age of an association between humans and *H. pylori* has not been elucidated, other than that it predates 60 kya.

**Figure 2:**
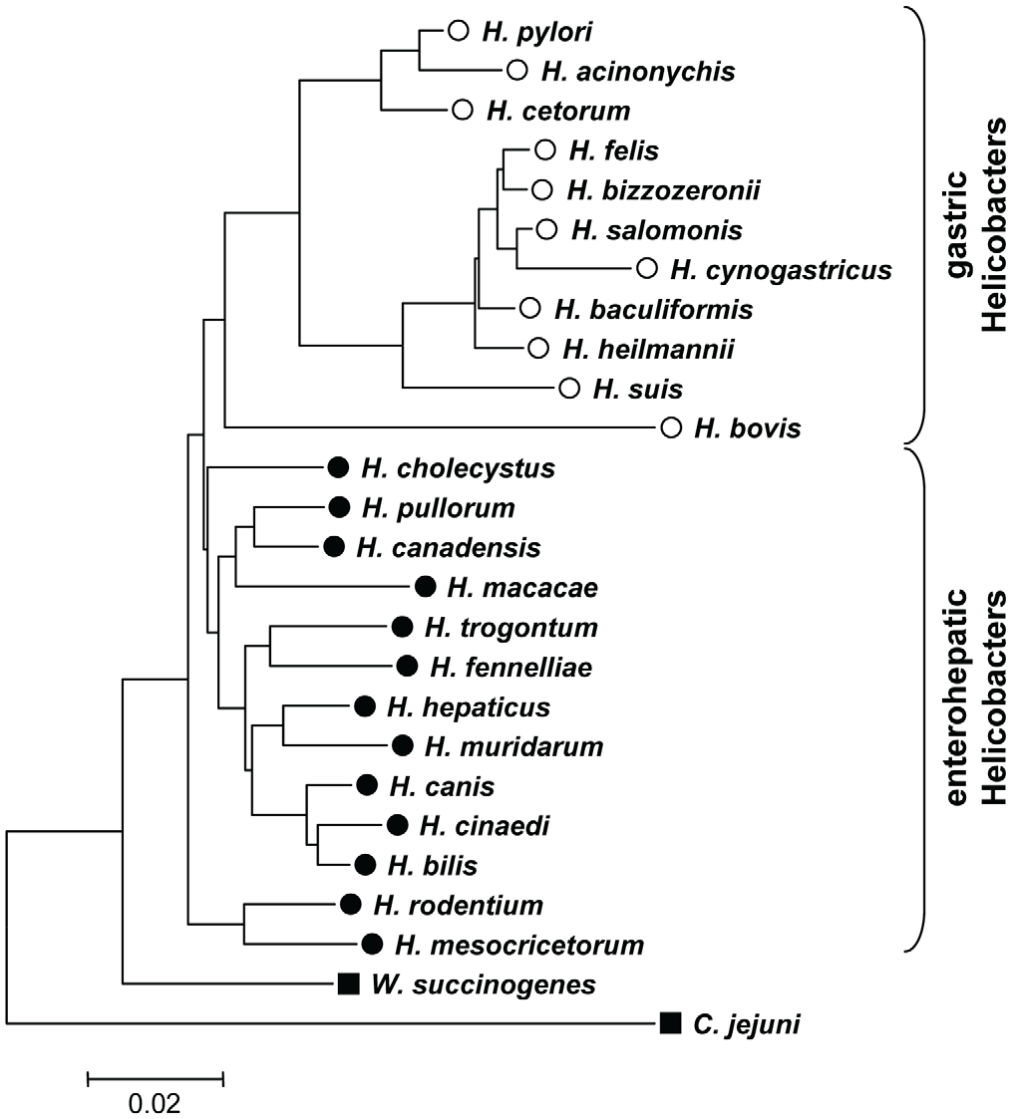
Neighbor-joining tree of 16SrRNA sequences from gastric and enterohepatic Helicobacter species. Sequences from gastric Helicobacters (open circles) such as *H. pylori*, *H. cetorum* or *H. suis* form a cluster separate from enterohepatic Helicobacter species (filled circles) such as *H. cinaedi* or *H. macacae*. The tree was rooted with the 16SrRNA sequences of the epsilon-proteobacteria *Wolinella succinogenes* and *Campylobacter jejuni* (squares). 16SrRNA sequences were obtained from Genbank, accession numbers are provided in Materials and Methods.

One possible scenario is that *H. pylori* has infected humans since their origins, possibly even prior to the origins of anatomically modern humans. In that event, we might expect to find *H. pylori*-like bacteria infecting our closest extant relatives, chimpanzees (*Pan troglodytes*), with whom humans shared a common ancestor ca. 5.4 million years ago (mya) [12]. We tested this hypothesis with negative results, suggesting that human infection by *H. pylori* likely post-dated the evolution of humans and resulted from a host jump from a different animal. Host jumps are not necessarily unlikely, because the stomachs of multiple animals are infected by diverse *Helicobacter* species, whose phylogeny is incongruent with that of their hosts [13]. Indeed, the closest known relative of *H. pylori* is *H. acinonychis*, which infects large felines and seems to have arisen by a host jump from humans [14]. And the next closest relative is *Helicobacter cetorum*, which infects dolphins and whales (Figure 2) [15]. All other *Helicobacter* species are genetically much more distinct.

**Figure 3:**
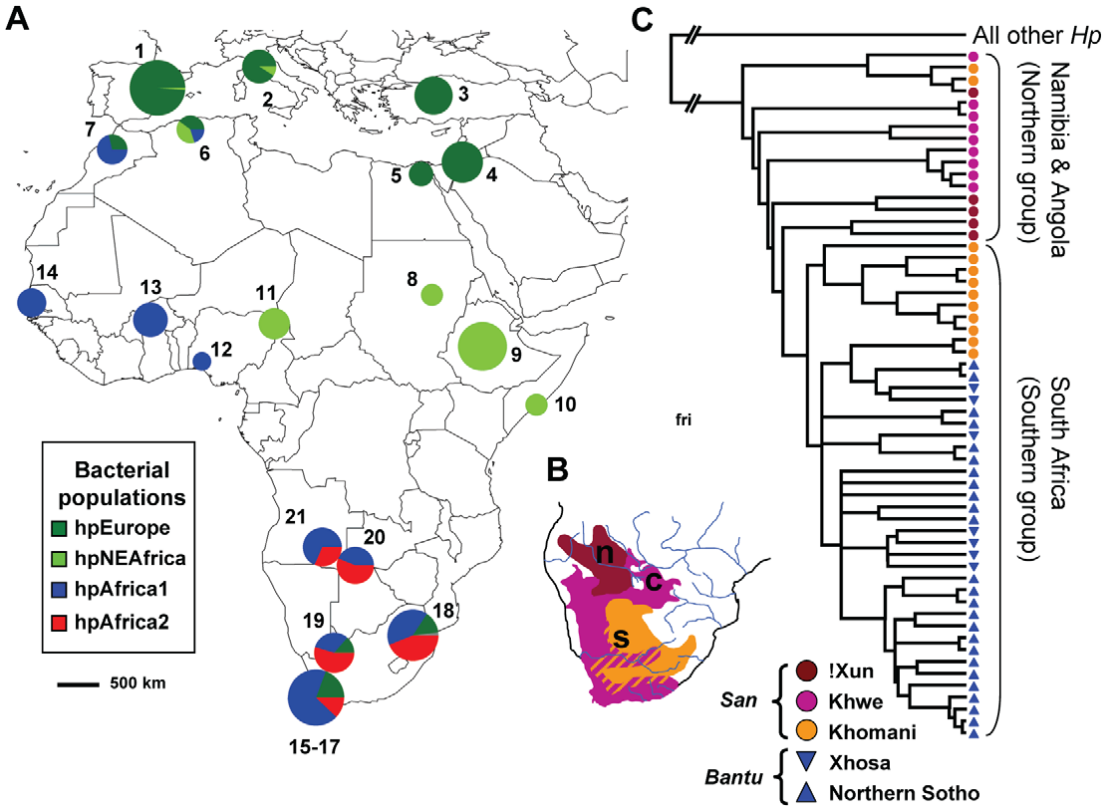
The distribution of *H. pylori* populations in Africa. (A) The proportions of haplotypes at each sampling location (numbers; Table 2, Table S1) from different bacterial populations are displayed as pie charts whose sizes indicate the numbers of haplotypes. (B) The distribution of the three major subgroups of the San language family in south-central Africa (adapted from [21]). s: Southern Khoisan was spoken on much of the South African plateau and the central Kalahari in Botswana; c: Central Khoisan was distributed in southern and western South Africa, most of Namibia and most of northern Botswana; and n: Northern Khoisan (Ju), was spoken in southern Angola, north-eastern Namibia and north-western Botswana (Table 1). The position of the letters indicates the geographical origin of Northern San (n; !Xun from Angola), Central San (c; Khwe from Namibia) and Southern San (s; Khomani from South Africa). (C) Phylogenetic relationships among hpAfrica2 strains (80% consensus of 100 ClonalFrame analyses). The tree was rooted with *H. pylori* strains from other populations.

If *H. pylori* infection of humans reflected a host jump prior to 60 kya, genetic traces of that event might be present within populations of *H. pylori* that are native to Africa, where modern humans originated. However, those populations have not yet been extensively sampled, except in West Africa or among recent migrants to South Africa. Diversity within human mitochondrial DNA (mtDNA) coalesces to a last common ancestor ca. 200 kya [16], [17], the time of divergence of mitochondrial haplogroup L0 from haplogroups L1–6. Haplogroups L0–6 are restricted to Africans, whereas three sub-haplogroups of L3 (haplogroups M, N and R) are found globally. L0d, the earliest sub-division within L0, and L0k, are particularly frequent among the San [16], [18], [19], indigenous people who pursued a nomadic, hunter-gatherer lifestyle until very recently. The San are thought to have been originally distributed throughout large parts of central and southern Africa, but are currently restricted to southern Africa [16], [18], [20]. The San speak variants of the “click language” Khoisan, one of the most ancient human language families, currently consisting of three language sub-groups which were geographically distinct within south-central Africa during pre-colonial times: Northern, Central and Southern Khoisan (Figure 3B) [21]. Around 5,000 years ago the Bantu people, consisting of agriculturalists from southern Nigeria and Cameroon who spoke dialects of Niger-Congo, expanded eastwards and southwards into regions of sub-equatorial Africa that were suitable for their equatorial crops [22]. Bantu populations replaced and/or absorbed most of the original, indigenous hunter-gatherer societies in Africa, and their expansion reached its southern limit in eastern South Africa around 700 AD. During that expansion, mtDNA lineages from the indigenous populations such as the San were assimilated into the Bantu gene pool, which is otherwise predominantly composed of L2 and L3 mtDNA haplogroups [23]–[25]. The San continued to thrive in regions that were even further south and west, which were climatically unsuitable for Bantu agricultural crops, such as modern South Africa, Namibia, Botswana and southern Angola. However, the San have been largely displaced since the arrival of Europeans in the 15^th^ century.

We hypothesized that the San might host descendents of the most ancient *H. pylori* populations, but until now San have not been screened for *H. pylori* infection. In particular, we anticipated that they might be infected by hpAfrica2, because that population has previously only been isolated in South Africa and it is very distinct from all other *H. pylori* populations (Figure 1) [2], [6], [7], [26]. We therefore isolated *H. pylori* from San individuals and calculated the divergence time (TMRCA) when these bacteria split from other African isolates in order to estimate the minimum age of an association between *H. pylori* and humans.

## Results

### *H. pylori* from San

Duplicate gastric biopsies from antrum and corpus of the stomach and peripheral blood samples were obtained from 30 San volunteers from the !Xun, Khwe and Khomani communities, which represent all three Khoisan language sub-groupings (Table 1). Similar to previous mtDNA genotyping of 31 DNAs from San [16], [19], our analysis of the blood samples showed that mtDNA haplogroups L0d and L0k are particularly frequent in San (67%), much more frequent than in Bantu (Northern Sotho) from South Africa (25%; *p* = 0.024, U-test) (Table 2). We also cultivated 131 *H. pylori* isolates from the San biopsies (Table S1), from which we sequenced the same seven housekeeping gene fragments that had previously been used for global analyses [7]. These sequences were concatenated to yield haplotypes with a total length of 3,406 base pairs, of which 56 were unique. Many duplicate haplotypes were obtained from multiple colonies within individual donors, as expected by clonal expansion from a single source. Identical haplotypes were also found between three pairs of donors, suggesting recent transmission (Table S1). The 56 unique haplotypes were distinct from 234 other haplotypes from Africa and 133 from Europe or the Middle East (Table 2).

**Table 1:** Origin of the San samples.

Community	Country	Original geographic distribution	Language group
Khomani	South Africa	South African plateau and central Kalahari desert in Botswana	Southern Khoisan (Tuu)
Khwe	Namibia	southern and western South Africa, most of Namibia and most of northern Botswana	Central Khoisan (Khoe-Kwadi)
!Xun	Angola	southern Angola, north-eastern Namibia and north-western Botswana	Northern Khoisan (Ju)

The individuals from the Khwe and !Xun communities in this study had recently migrated to South Africa.

**Table 2:** Population assignment of unique *H. pylori* haplotypes from Africa/Mediterranean and human mtDNA haplogroups from Southern Africa.

ID	Country/Area	Ethnic origin	*Helicobacter pylori* population					Human mitochondrial haplogroup^1^									
			hpAsia2	hpEurope	hpNEAfrica	hpAfrica1	hpAfrica2	L0a	L0d	L0k	L1c	L2a	L2b	L3d	L3e	L3f	Totals
1	Spain		0	79	1	0	0										
2	Italy		0	10	1	0	0										
3	Turkey		0	18	0	0	0										
4	Middle East		0	24	0	0	0										
5	Egypt		0	3	0	0	0										
6	Algeria		0	2	2	1	0										
7	Morocco		0	2	0	5	0										
8	Sudan		0	0	2	0	0										
9	Ethiopia		0	0	48	0	0										
10	Somalia		0	0	2	0	0										
11	Nigeria/Maiduguri	Kanuri	0	0	8	0	0										
12	Nigeria/Lagos	Yoruba	0	0	0	1	0										
13	Burkina Faso		0	0	0	12	0										
14	Senegal		0	0	0	6	0										
15	South Africa/Cape Town	Cape Coloured	0	5	0	32	0										
16	South Africa/Cape Town	European	0	9	0	8	3										
17	South Africa/Cape Town	Xhosa (Bantu)	0	2	0	17	7										
18	South Africa/Pretoria	Northern Sotho (Bantu)	1	8	0	23	25	21	30	2	6	33	1	23	12	1	129
19	South Africa	Khomani San	0	3	0	7	12	0	8	0	0	0	0	0	1	0	9
20	Namibia	Khwe (San)	0	0	0	5	9	0		2	0	1	0	1	4	0	8
21	Angola	!Xun (San)	0	0	0	13	5	0	7	2	0	0	0	0	2	0	11
	Southern Africa	San (Unknown ethnicity)	0	0	0	2	0	0	0	1	0	0	0	0	1	0	2
**Totals**			1	165	64	132	61	21	45	7	6	34	1	24	20	1	

1 mtDNA haplogroups were not determined for samples 1 to 17.

**Figure 4:**
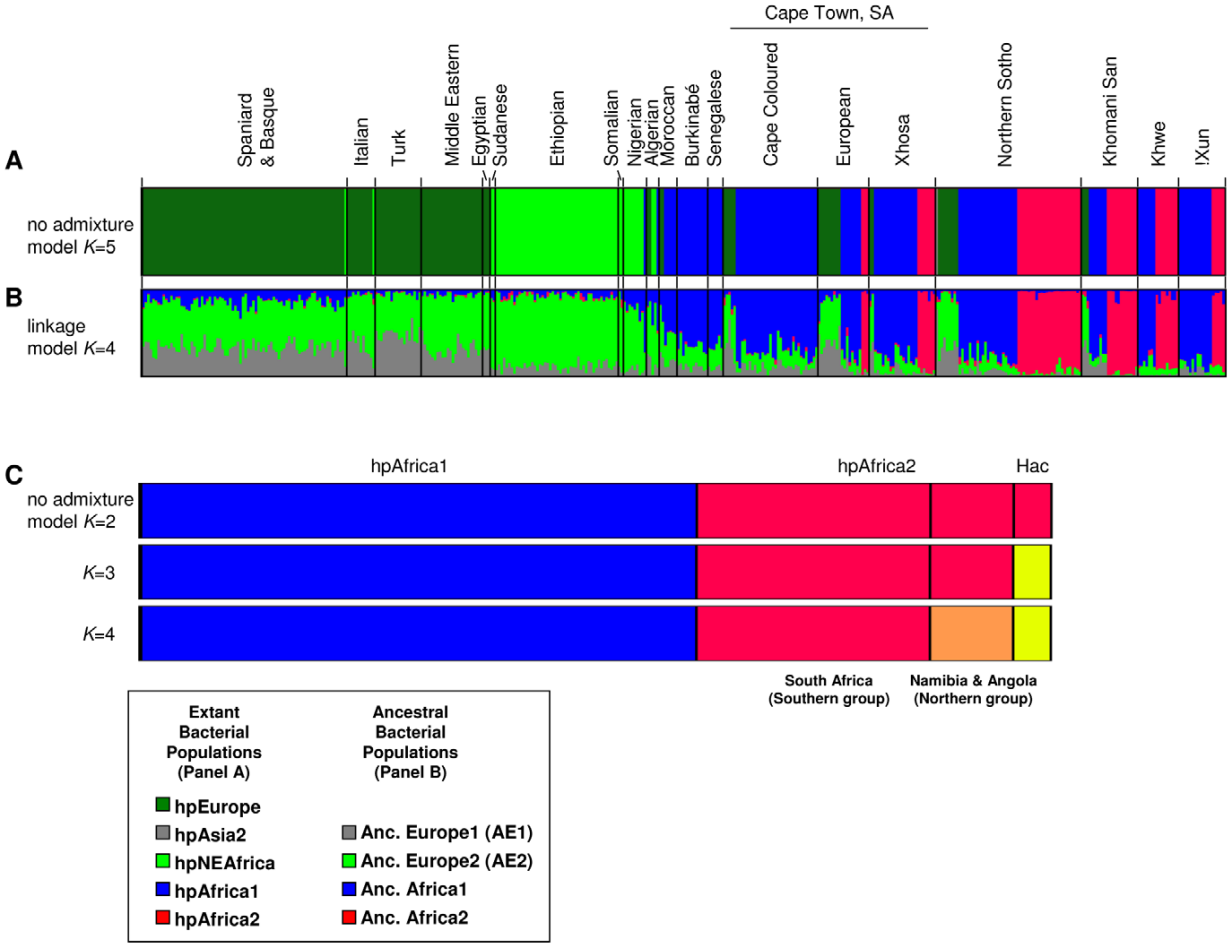
Bayesian population assignments using Structure V2.0. (A) Distruct plot of the assignment of *H. pylori* haplotypes from Africa, the Middle East and southern Europe as determined by the no admixture model. Each isolate is represented by a thin line that is color coded according to the population assignment. (B) Distruct plot of the proportions of ancestral nucleotides as determined by the linkage model. A thin line for each isolate indicates the estimated amount of ancestry from each of the four ancestral populations as four colored segments. (C) Distruct plot of the population assignment (no admixture model) of *H. pylori* hpAfrica1 and hpAfrica2 haplotypes from southern Africa and *H. acinonychis* (Hac).

In order to assign *H. pylori* from San to populations, we combined these 56 unique haplotypes with 83 haplotypes from two Bantu ethnic groups in South Africa (Northern Sothos living in the Mpumalanga Province near Pretoria [2], [26], and Xhosa from Cape Town [6]), 37 haplotypes from Cape Coloured in South Africa, 91 haplotypes from other areas of Africa and 133 from Europe and the Middle East (Table 2, Figure 4A,B). (In other analyses (data not shown) we also included a global reference data set of 1040 haplotypes that had previously been assigned to *H. pylori* populations [7], [8] but they consistently yielded the same population assignments for the reference haplotypes as in previous studies, and did not reveal any novel populations.) Bayesian cluster analysis was performed with the non-admixture model of Structure [5] for estimates of the total number of populations, K, between 2 and 5, which was the highest value of K that yielded consistent clustering and consistent probability estimates between individual runs. Almost half of the San haplotypes (26/56, 46%) belong to hpAfrica2 (Figure 3A, Figure 4A,B, Table 2). hpAfrica2 isolates were found in all three San communities, ranging in frequency from 28% of all haplotypes (!Xun) to 55% (Khwe, Khomani).We also identified 35 hpAfrica2 haplotypes among isolates from the Northern Sotho near Pretoria and from Xhosa and Europeans in Cape Town.

hpAfrica2 is related to *H. acinonychis* (Hac) from large felines (see below). We therefore performed additional Structure analyses on haplotypes of hpAfrica2, hpAfrica1 and Hac. Under a two population model (*K* = 2), hpAfrica2 clustered together with Hac, separately from hpAfrica1 (Figure 4C). hpAfrica1, hpAfrica2 and Hac were all distinct at *K* = 3 whereas at *K* = 4, hpAfrica2 haplotypes partitioned into two sequence clusters, one associated with southern isolates from South Africa (Khomani and non-San) and the second with northern isolates from speakers of the Northern Khoisan (!Xun) and Central Khoisan (Khwe) language subgroups (Figure 3C, Figure 4C). The only exceptions were two haplotypes from one Khomani San individual, which were assigned to the northern isolates.

The level of genetic diversity, π, was significantly higher among hpAfrica2 haplotypes from San (95% CL [2.83,3.08%]) than from Bantus ([2.52,2.80%]), suggesting that the San isolates might be ancestral. To test this inference, we determined the phylogenetic structure of all 58 hpAfrica2 haplotypes with ClonalFrame, which can discern ancestral relationships even in the presence of homologous recombination [27], [28] (see below). The consensus tree from this analysis shows that the southern (Khomani, Bantu) San haplotypes fell into a young clade which emerged from an more ancestral population of hpAfrica2 haplotypes, all of which were from San and most of which were from the northern Khwe and !Xun (Figure 3C). These observations suggest that hpAfrica2 evolved within the San and was subsequently transmitted to Bantus.

Almost all non-other haplotypes from San were assigned to hpAfrica1. In contrast, to the results described above, these were less diverse (π 95% CL [2.50, 2.82%]) than hpAfrica1 from Bantus ([3.10, 3.20%]), suggesting that the San had acquired hpAfrica1 from Bantu.

### Age of the association of *H. pylori* and humans

**Figure 5:**
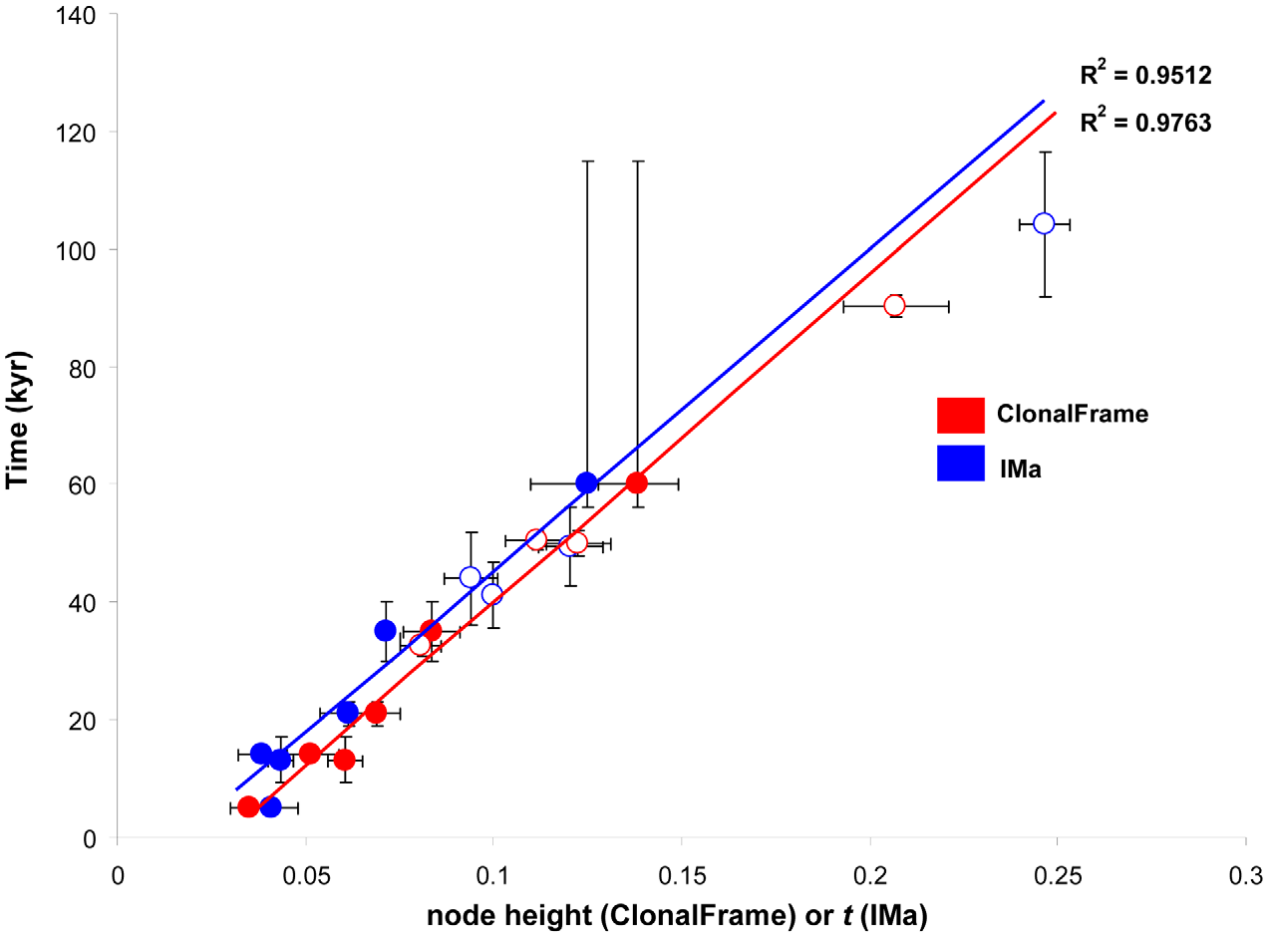
Linear relationships between known calibration dates and posterior parameters node height (ClonalFrame) and *t* (IMa). Circles in red show ClonalFrame estimates and those in blue are IMa values. Closed circles denote median values for calibration times used for the regression (Table 3). Open circles show inferred times for the four African pair-wise comparisons (Table 4) after calibration with rate-smoothing.

The ages of lineages of closely related bacteria that evolved in recent decades can be dated by genomic analyses of isolates known to span that time range [29], [30]. However, it is difficult to accurately date the origins of individual lineages or species of microorganisms over longer time periods [31]. For example, although HIV is thought to be of recent origin, rabbit retroviruses that are related to HIV differentiated from them over seven million years ago [32], raising the possibility that HIV itself is also old but that its diversity was reduced during a recent bottleneck. We have developed a method for dating the origins of *H. pylori* in the last 60 kyr by calibrating the genetic distances between *H. pylori* population against the dates of separation of the corresponding human populations [8]. After stripping signals introduced by homologous recombination from the *H. pylori* sequence data, a linear relationship was found between the genetic distances and the archaeological dates (Figure 5), which allowed the estimation of unknown dates of population splits, such as the original peopling of the Pacific [8]. We have now used this approach to calculate the age of splits between the African lineages of *H. pylori* plus Hac. Similar to our previous analyses, we used two independent approaches to construct a phylogenetic tree, ClonalFrame [27] and IMa) [33].

ClonalFrame calculates a coalescent whose branch lengths exclude stretches of clustered nucleotide polymorphisms that result from recombination, although these stretches are used to calculate the topology when they are informative [27]. ClonalFrame can retrieve the clonal frame in moderately recombining bacteria such as *Bacillus* and *Salmonella* [27], [34]. And in our experience, the phylogenies recovered by ClonalFrame for *H. pylori* are quite insensitive to the size of the dataset. However, determining an accurate age for the coalescent with such an approach depends on accurate rooting through an outgroup. We were unable to accurately root the tree with housekeeping genes from published genomes of enterohepatic *Helicobacter* species or from *Campylobacter* species because none of them contained orthologs of all seven housekeeping genes in our dataset. We therefore shotgun sequenced the genome of *H. cetorum* strain MIT 99-5665, which represents the closest known relative of *H. pylori* and Hac [15] (Figure 2), and used the orthologous nucleotide sequences from that genome as an outgroup for rooting the ClonalFrame tree. Independent analyses yielded the same rooting branch point when the tree was rooted with and based on orthologs that were shared between *H. pylori* and enterohepatic *Helicobacter* genomes (data not shown).

IMa is a mainstream method for the inference of historical population genetic parameters that were associated with historical splits between pairs of populations [33]. IMa simulates the posterior probabilities for the population parameters theta (Neμ), where Ne is the effective population size and μ is the mutation rate, m (the effective number of migrants per generation) and *t* (the time since population splitting). IMa accounts for intermittent back-migration after population splits. We therefore identified blocks of recombinant DNA in each pair of populations by the four-gamete test [35] and stripped those blocks from the data. This test assumes an infinite sites model, which is only applicable when the mutation rate is lower than the recombination rate, as is the case for *H. pylori* [4]. The remaining blocks of sequence were used to estimate dates of splitting by the isolation with back-migration model. Although we do not know of other attempts, except our own [8], to use IMa for the dating of bacterial phylogenies, it has been extensively used for to date population splits among eukaryotic populations [36]–[38].

**Figure 6:**
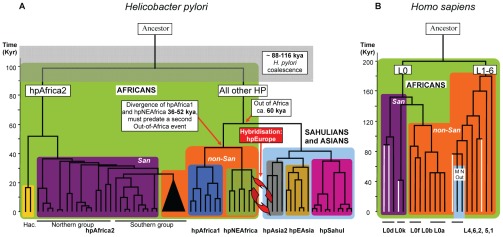
A comparison of global *H. pylori* and human mtDNA phylogenies. (A) Global phylogeny of *H. pylori* displayed as the strict consensus of 100 ClonalFrame analyses. After outgroup-rooting with *H. cetorum*, the time of the basal *H. pylori* divergence between hpAfrica2 and all other populations was estimated to 102 kya (95% confidence limit, 88–116 kya). Divergence of the other African *H. pylori* populations, hpAfrica1 and hpNEAfrica, began between 36 and 52 kya. (B) Simplified human mtDNA phylogeny adapted from Behar *et al.*, 2008 [14] with the permission of *AJHG*. African lineages are shown on a green background whereas the background for lineages outside Africa is light blue. San clades are purple, non-San clades are orange and *H. acinonychis* is yellow. San mtDNA lineages in our sample are shown as white lines.

**Figure 7:**
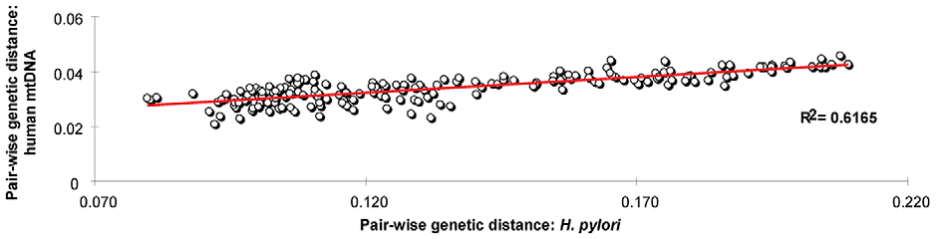
Parallel patterns of pair-wise genetic distances between human mtDNA and *H. pylori* gene sequences. A Mantel regression (*R* ^2^ = 0.62, P<0.0001) showed that 62% of the pair-wise genetic distances between *H. pylori* sequences can be accounted for by the pair-wise genetic distances between human mtDNA sequences from analogous geographic locations.

The models used by ClonalFrame and IMa are fundamentally different, except that both used the same archaeological and molecular calibration points (Table 3). A linear relationship between genetic distance and calibration date with high regression coefficients was found by both sets of analyses (Figure 5), and they estimated overlapping extrapolated dates, with one minor exception (Table 4). These overlapping estimates indicate that our age estimates are primarily dependent on the archaeological calibrations and are independent of method. The TMRCA of all *H. pylori* plus Hac lineages was 88–116 kya (ClonalFrame: 88–92 kya; IMa: 92–116 kya; Table 4, Figure 6A). The date for the coalescence of non-recombining Y-chromosome lineages in modern humans is similar at 90 kya [39] to 141.5±15.6 kya [40] whereas the date of split between L0 and L1–6 mtDNA haplogroups in humans is older, 194.3±32.5 kya, (Figure 6B) [16], [17]. Despite the different age estimates, the topology and branching pattern of the genealogies are strikingly similar between *H. pylori* and human mtDNA (Figure 6). The similarity between these two trees could not be compared directly because the numbers of lineages differ between the two genealogies. We therefore performed a quantitative test of whether similar phylogeographic trends exist in both *H. pylori* and mtDNA data by performing a Mantel regression of the maximum composite likelihood distances between pairs of populations from comparable geographic sources of both humans and *H. pylori* (Figure 7, Tables S3, S4). The results showed that 60% of the variation in both data sets is distributed similarly (P<0.0001).

**Table 3:** Known human population events inferred from either archaeological or molecular studies and used to calibrate dating analyses.

Demographic Event	*H. pylori* populations used for dating		Calibration time range (kya)	Evidence	Reference
	Population 1 (n1)	Population 2 (n2)			
Out of Africa	hpNEAfrica & hpAfrica1 (54)	All non-African *H. pylori* (35)	56–115	*H. pylori*, mtDNA, microsatellites	[7], [42], [86]
Split between Central and East Asia	hpAsia2 (50)	hpEastAsia (50)	30–40	Archaeology	[87]
Peopling of the Americas	hspEAsia (18)	hspAmerind (18)	19–23	mtDNA	[88]
First humans in Taiwan	hspEAsia (38)	hspMaori (65)	9.4–17	mtDNA	[89]
Start of the Austronesian Expansion	hspMaori Taiwan (23)	hspMaori Pacific (44)	4.9–5.0	Archaeology	[90], [91]
Expansion to South America	hspAmerind North America (17)	hspAmerind South America (7)	13.98–14.22	mtDNA	[92]

n, number of haplotypes used in pair-wise IMa simulations.

kya, kilo years ago.

hp, *Helicobacter* population.

hsp, *Helicobacter* sub-population.

hspAmerind South America, South American subclade within hspAmerind.

hspAmerind North America, North American subclade within hspAmerind.

hspMaori Taiwan, the subgroup of hspMaori isolated in aboriginal people of Taiwan.

hspMaori Pacific, a derived subclade within hspMaori consisting of isolates from the Philippines, Melanesia, Polynesia and New Zealand.

**Table 4:** Posterior population parameters simulated using an isolation with migration model (IMa) and ClonalFrame for African *H. pylori* populations.

			Theta		Migration parameter		Migration rate/gen (m = θ*m*)		*t* (IMa)		Node height (ClonalFrame)		TMRCA (CL 95, kya)	
	Population 1 (n1)	Population 2 (n2)	θ1	θ2	*m*1	*m*2	m1	m2	Lower	Upper	Lower	Upper	IMa	ClonalFrame
1	hpAfrica2 (42)	All other *H.pylori* (42)	0.054	0.138	13.628	2.232	0.737	0.308	0.240	0.253	0.193	0.221	92.0–116.4	88.4–92.3
2	*H. acinonychis* (7)	non-*H. acinonychis* hpAfrica2 (28)	0.056	0.237	0.000	0.000	0.000	0.000	0.112	0.129	0.114	0.131	42.8–56.2	47.7–52.1
3	hpAfrica2 Southern Group (18)	hpAfrica2 Northern Group (16)	0.162	0.023	2.759	97.515	0.448	2.194	0.099	0.101	0.075	0.086	35.6–46.8	31.6–33.4
4	hpNEAfrica (26)	hpAfrica1 (28)	0.126	0.047	7.990	41.390	1.008	1.929	0.087	0.101	0.103	0.120	36.2–51.9	49.0–52.2

gen, generation.

CL 95, 95% confidence limit.

n, number of haplotypes used in pair-wise IMa simulations.

kya, kilo years ago.

hp, *Helicobacter* population.

hsp, *Helicobacter* sub-population.

hpAfrica2 Southern Group, derived subclade of hpAfrica2, isolated mainly in San and Bantu-speakers of South Africa.

hpAfrica2 Northern Group, subgroup of hpAfrica2, isolated in San inhabitants of Namibia and Angola.

TMRCA, time to the most recent common ancestor.

### Dates of internal splits between *H. pylori* populations

We also used these data to estimate the ages of splits between individual lineages within the *H. pylori*/Hac phylogeny, all of which seem to be later than the Out of Africa migrations of 60 kya [7]. Descendents from the last common ancestors of *H. pylori* plus Hac diverged into two distinct super-lineages, one of which gave rise to hpAfrica2 plus Hac and the second of which gave rise to all other populations (Figure 6A). The TMRCA for the split between hpAfrica2 and Hac is 43–56 kya (Table 4), and hpAfrica2 subsequently split (32–47 kya) into the northern and southern isolates. We note that a similar date (40 kya) was recently estimated for the TMRCA of Y-chromosome haplogroup A-M51 among the San by Henn *et al.* [20], which also subsequently split between northern and southern San populations. Within the other super-lineage, the estimated TMRCA was 36–52 kya for the African populations hpAfrica1 and hpNEAfrica (Table 4).

### Absence of *H. pylori* in wild-born chimpanzees

Our calculated TMRCA of 88–116 kya for *H. pylori* plus Hac might represent the date of a host jump to humans from a different animal host. Due to lineage sorting and bottlenecks, the date of such a host jump may also have been considerably earlier. We therefore attempted to isolate *H. pylori* from chimpanzees, who are our closest relative. We collected stomach antrum and corpus biopsies from 42 captive, wild-born chimpanzees (*Pan troglodytes*) that originated from the Great Lakes region of Central-East Africa (Uganda, Rwanda and the Democratic Republic of the Congo) but now live on an isolated island sanctuary in Uganda. Endoscopic examination during esophagogastroduodenoscopy identified a mild gastritis in some animals, suggesting that they might be infected with *H. pylori*. However it is known that there is a poor correlation between the endoscopic presence of gastritis and the prevalence of *H. pylori* as gastritis may also be caused by other non-infectious etiologies. The biopsies were taken with single use biopsy forceps (Radial Jaw, Boston Scientific) by an experienced gastroenterologist who routinely obtains aseptic biopsies that allow cultivation of *H. pylori*, including hpAfrica2. The biopsies were immediately stored and transported in liquid nitrogen. Attempts at cultivation of the biopsies were performed in an H_2_-containing microaerophilic atmosphere under growth conditions that are routinely successful for the cultivation of *H. pylori*, *H. acinonychis* and *H. cetorum*. Over the past years, these methods have successfully cultivated over one thousand *H. pylori* strains from multiple geographic locations including remote regions of Siberia, Papua New Guinea and Cameroon. And they routinely succeed with hpAfrica2, which are particularly difficult to grow in the absence of atmospheric H_2_ (unpublished data). However, we were unable to cultivate *Helicobacter*-like bacteria from any of the chimpanzee biopsies.

We reasoned that *H. pylori*-like bacteria from chimpanzees might not grow under these cultivation conditions, and therefore attempted to amplify 16S rRNA sequences from the bacterial DNA in the biopsies. To this end, we designed oligonucleotide primers that should successfully amplify PCR from any *Helicobacter* species. In independent (unpublished) experiments, these primers have been successful at amplifying rRNA sequences from *H. cetorum* in fecal samples from dolphins. However, the PCRs performed with DNA extracted from the chimpanzee biopsies all failed to amplify any products, except for two instances of air contamination from previous water controls. These results suggest that *H. pylori* might be rare in chimpanzees, possibly indicating that it has not coevolved with hominids during the evolution of the great apes.

## Discussion

### Human source of hpAfrica2

This project was initiated because we were intrigued by the great genetic distance between hpAfrica2 and other populations of *H. pylori* as well as by the geographical distribution of hpAfrica 2, which has only been isolated in South Africa. Our data indicate that both of these observations result from an original association between hpAfrica2 and the San. In support of this interpretation, the deepest branches within the hpAfrica2 genealogy are associated with the northern San, represented by the !Xun and Khwe ethnic communities (Figure 3). A further indication that hpAfrica2 evolved in the ancestors of these northern click-speaking people are the results obtained with IMa, according to which migration within the hpAfrica2 lineage has been predominantly from north to south (m2 = 2.20) rather than south to north (m1 = 0.45) (Table 4). Finally, the genetic diversity is greater among hpAfrica2 from San than from Bantu, indicating that it was transmitted to Bantu in the last few hundred years since their arrival in southern Africa.

These conclusions are also relevant to the host jump from humans to large felines which gave rise to *H. acinonychis* (Hac) [14]. Firstly, our population assignments and phylogenetic reconstructions show that although they are discrete taxonomic species, Hac is part of the same genetic super-lineage of *H. pylori* as hpAfrica2 (Figures 4 and 6A), and the host jump occurred after *H. pylori* had sub-divided into two super-lineages, The coalescence of hpAfrica2 and Hac was estimated at 43–56 kya, which provides an estimate of the date of the host jump to large felines. This is later than our prior estimate of the date of that host jump as 100 kya [14], [41]. However, Hac was thought to be phylogenetically distinct from *H. pylori*, rather than nested within it, and we based our calculation on a comparison of the genomes of strains 26695 (hpEurope), J99 (hpAfrica1) and Sheeba (Hac), which essentially equates to the coalescent for the two super-lineages of *H. pylori* of approximately 100 kya. In the light of our conclusion that the hpAfrica2-containing super-lineage is associated with the San, the host jump that resulted in Hac may have arisen after the consumption of the stomach contents of an infected ancestor of the San by a large feline.

### Length of association of *H. pylori* with humans

Our data shows that anatomically modern humans were infected by *H. pylori* long before their migrations out of Africa of ∼60 kya [7], [42]. We estimate the minimum age of that association to be approximately 100 kyr (range 88–116). This is comparable to the age of the coalescence of the human Y-chromosome and about half of the coalescent for mtDNA. The age of a coalescent is a minimal date estimate because lineage sorting and bottlenecks lead to extinction of older lineages, resulting in a single genealogical source of all subsequent descendents. Indeed, the genealogies of *H. pylori* and mtDNA are very similar (Figure 6), and a Mantel regression indicated that the geographical distribution of the genetic diversity within both humans and *H. pylori* is also similar, both within Africa and outside. These results suggest that anatomically modern humans were infected by *H. pylori* since their origins. We therefore anticipated that we would isolate relatives of *H. pylori* from wild-born chimpanzees, our genetically closest relatives. However, we failed in this effort, and also failed to PCR amplify *Helicobacter* rRNA sequences.

Our failure does not provide convincing evidence that chimpanzees are not infected with close relatives of *H. pylori*. Those close relatives might not have been capable of growth under the conditions we used. Alternatively, unknown technical problems might have affected the sampling, transportation or PCR reactions. We only sampled 42 chimpanzees of subspecies *Pan troglodytes schweinfurthii*, all of whom were from East and Central Africa, and chimpanzees elsewhere in Africa might be infected with a *H. pylori*-like organism. We also note that the lack of isolation of *Plasmodium* spp. from eastern lowland gorillas and bonobos [31] and SIV in eastern chimpanzees [43] was due to variable infection rates among hominid apes from different areas of Africa. And as a final alternative, the human-chimpanzee ancestor might have been infected with *Helicobacter* precursors, but chimpanzees subsequently lost those bacteria secondarily. Additional analyses involving the other chimpanzee subspecies as well as bonobos and gorillas might help resolve these uncertainties.

The literature contains many reports of the isolation of *H. pylori* from distantly related primates. These probably reflect either transmission from humans to animals during captivity, or infection with genetically distantly related *Helicobacter*. *H. pylori*-like bacteria have been isolated from macaques, including the named species *H. nemestrinae* [44]. However, the haplotype of *H. nemestrinae*, which was isolated from pigtailed macaques (*Macaca nemestrina*) in an American zoo, was subsequently shown to belong to the hpEurope population of *H. pylori*, which is common in the USA [45]. Widespread *H. pylori* infection has been reported among rhesus macaques (*Macaca mulatta*) from China [46] and crab-eating macaques (*Macaca fascicularis*) from Vietnam and the Philippines [47]. The 16S rRNA sequences of isolates from Philippine macaques were similar to those of hpEurope, which is also frequently isolated from inhabitants of the Philippines [7], [10]. Isolates from Vietnamese macaques belonged to hpEastAsia [47], as do most *H. pylori* strains from Vietnamese [7]. In fact, macaques are so readily colonized by human *H. pylori* that rhesus macaques [48], crab-eating macaques [49] and Japanese macaques (*Macaca fuscata*) [50] are all used as animal models for *H. pylori* infection and pathogenicity.

Other isolates of *Helicobacter* from primates are from species that are only very distantly related to *H. pylori*. In addition to *H. pylori* of presumptive human origin, macaques are also infected with *Helicobacter suis* [51], *Helicobacter cinaedi* [52] and *Helicobacter macacae* [53]. *H. suis* has also been isolated from mandrill monkeys and crab-eating macaques in a zoo [51]. However, *H. suis* is associated with gastritis and ulceration in pigs, and belongs to a parallel lineage to *H. pylori*, *H. acinonychis* and *H. cetorum* in a phylogenetic tree of 16S rRNA sequences (Figure 2). *H. cinaedi* and *H. macacae* are even more distinct from *H. pylori* (Figure 2), and belong to the genetically quite distinct enterohepatic *Helicobacters* whose primary site of infection is the intestine, colon or liver. Thus, none of these primate isolates are likely candidates for a close relative of *H. pylori* that might have co-evolved with hominid apes.

We conclude that there is no direct evidence for co-evolution of *H. pylori* and humans prior to approximately 100 kya. Furthermore, the genealogical relationships within *Helicobacter* 16S rRNA are consistent with multiple host jumps, as is already indicated by the fact that the closest relative of *H. pylori* are associated with large felines (Hac) and dolphins/whales (*H. cetorum*). We therefore propose that the association of *H. pylori* with humans also reflects a host jump to humans from an unknown species, which occurred approximately 100 kya or earlier. In principle, two later host jumps might explain the existence of two super-lineages of *H. pylori*, but this seems less likely because the similar phylogeographical patterns of *H. pylori* and mtDNA haplogroups indicate that they have undergone a parallel evolutionary history.

### Two ‘out of Africa’ migrations

Despite the general similarities between the genealogy of *H. pylori* and human mtDNA, there is a striking difference in respect to Europe.

Archaeological differences in the technology of stone tools have been used to justify two out of Africa migrations from two different source populations in Africa, the first spreading “Middle Paleolithic” technology in southern Asia, and the second distributing “Upper Paleolithic” from northern Africa into the Levant and Europe [54], [55]. However, a single successful out of Africa event is indicated by the fact that modern Asian and European mtDNA haplotypes are all derived from a subset of the L3 haplogroup [56], and two independent migrations from Africa were thought to be unlikely due to the greater diversity of mtDNA haplotypes in Africa [17], [25], [57].

**Figure 8:**
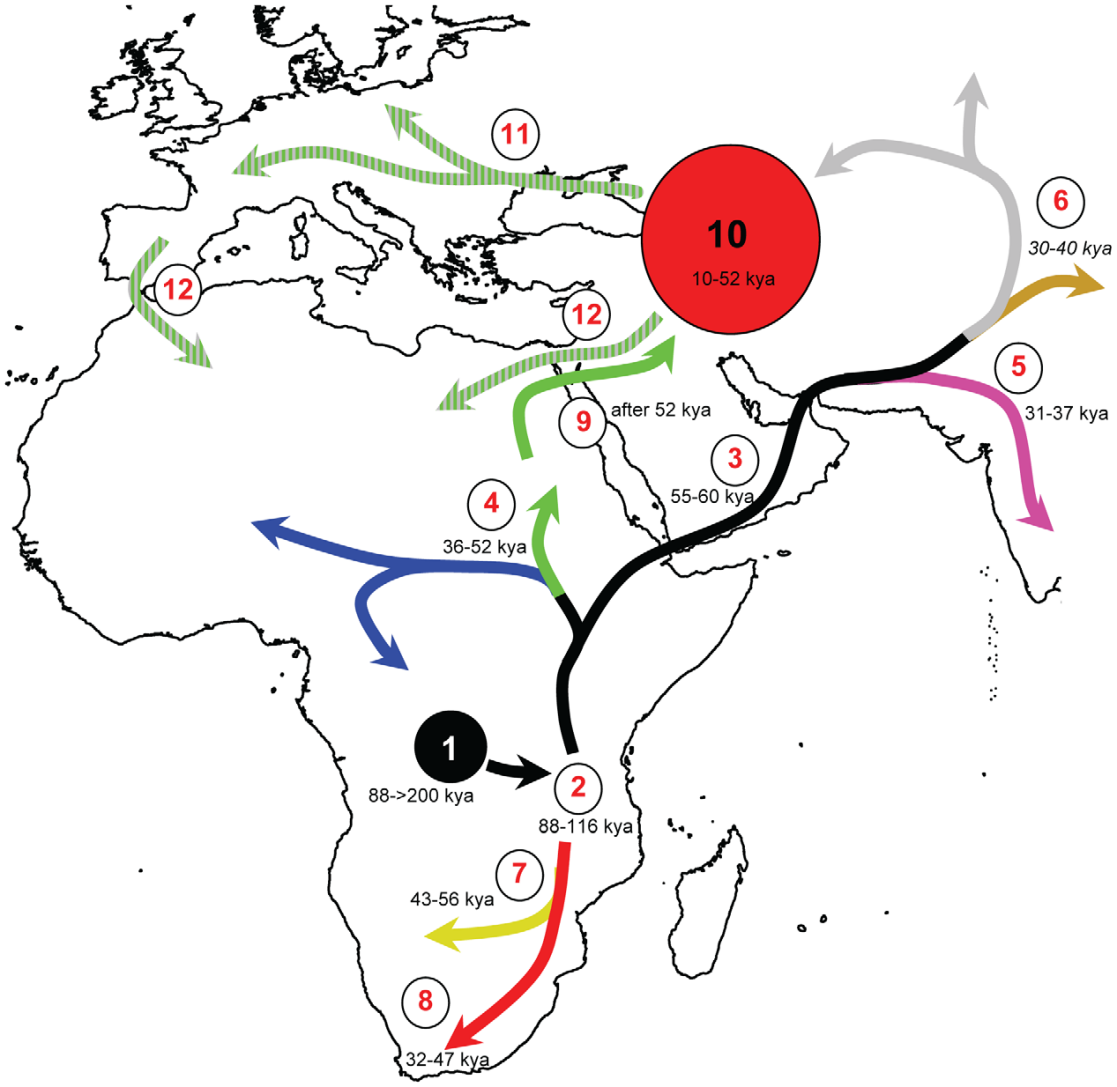
Chronological reconstruction of the major population events occurring during the intimate human-*H. pylori* association. Black lines indicate undifferentiated populations and all other lines are color-coded according to population as in Figs. 1, 3A, 4A, 6A. The sequence of events is as follows: 1) Initial acquisition of *H. pylori* by a human ancestor; 2) Divergence of *H. pylori* into two super-lineages; 3) First successful migration of modern humans Out of Africa [7], [42] via the southern route [57]; 4) *H. pylori* divergence into hpAfrica1 and hpNEAfrica with migration eastwards (hpNEAfrica) and westwards (hpAfrica1); 5) Divergence of *H. pylori* out of Africa into hpSahul [8] and 6) hpAsia2 and hpEastAsia; 7) Host jump from San to large felines giving rise to *H. acinonychis*. 8) Southward migration of San [16] carrying the ancestor of hpAfrica2; 9) Second successful migration Out of Africa via the Levant; 10) Hybridization of AE1 from central and south-west Asia and AE2 from north-east Africa [7] in the Middle East or western Asia resulting in hpEurope; 11) Spread of hpEurope bacteria to Europe; 12) Back migration from the Middle East [75], [76] and Spain [74] spreading hpEurope into North Africa. Dates in italics represent estimates obtained from sources other than *H. pylori*.

The phylogeographic diversity within *H. pylori* is inconsistent with a single human expansion from Africa. *H. pylori* accompanied humans on the migration of ∼60 kya [7], reaching Oceania not long thereafter [8]. However, European *H. pylori* possess distinct properties from most other global populations of these bacteria. *H. pylori* from Europe, the Middle East, western Asia and India belong to the hpEurope population [6], [7], [10], [58]–[60], which unlike Europeans is typified by great genetic diversity, greater than in Africa except for southern Africa where strong genetic diversity results from the presence of the second super-lineage (hpAfrica2). The great diversity of hpEurope was attributed to the fact that it is a hybrid population which arose from the admixture of AE1 (Ancestral Europe 1) and AE2 (Ancestral Europe 2) (Figure 4B) [6], [7]. AE1 arose in Central Asia after *H. pylori* was carried out of Africa during the Out of Africa migration of ∼60 kya [7], and its descendants are found among extant hpAsia2. However, the data in Figure 6A indicate that AE2, whose extant descendents in hpNEAfrica are associated with northeast Africa, first split from its sister lineage hpAfrica1 36–52 kya, after the (first) Out of Africa migration. We therefore hypothesize that a second Out of Africa migration in the last 52 kya brought AE2 to the Levant, after which it came into secondary contact with AE1. Subsequent extensive admixture resulted in hpEurope, which subsequently spread to Europe and western Asia (Figure 8). This interpretation differs from classical interpretations based on uni-parental markers (mtDNA, non-recombining Y chromosome) [56], [57] but a secondary colonization of Europe is supported by other archaeological and genetic data. Modern humans spread rapidly from the Levant to most of Europe by 40–46 kya [61]–[63], accompanied by “Upper Paleolithic” or “Mode 4” stone tools, which first occurred in North Africa and Eurasia after 50 kya [54], [63]. During the Last Glacial Maximum 26.5–19 kya [64], Europeans retreated to refugia such as the Iberian Peninsula and the Ukraine, which were the sources of re-colonization of Europe after the end of the ice age [65]–[68]. Signs of this re-colonization are evident in human DNA, e.g. mtDNA haplogroups that are wide-spread among Europeans (HV3, HV4, U4a1) can be traced back to 12–19 kya in eastern Europe, supporting an expansion from an Ukrainian glacial refugium [65]. Similarly, other common European haplogroups (V, H1, H3) arose in the northern Iberian peninsula soon after the Last Glacial Maximum, and dispersed into Europe after a population expansion in Iberia 10–15 kya [66]–[68].

The approach described here allowed estimates of the TMRCA of populations whose ancestry is largely derived from a single ancestral population, but does not allow dating of admixed populations such as hpEurope. hpEurope arose after its parental populations, i.e. within the last 52 kyr. Its near universal presence from Europe through to Western Asia may have been facilitated by any of multiple postulated major human migrations, including the initial colonization of Europe, the re-colonization of Europe from the Ukraine and Iberia and the Neolithic spread of agriculture from the Fertile Crescent into Europe, Western Asia and India in the last 10 kyr [22]. We note however that the migration of farmers from the Near East during the adoption of agriculture was likely very limited because the Near Eastern Neolithic component of the mtDNAs gene pool of modern Europeans is only 15% [69], and the majority of European lineages date back to late glacial and post-glacial times [70]. Similarly, ancient DNA analyses suggest that modern European ancestry is closer to that of the ancestral European hunter-gatherers than that of early farmers [71], [72]. If the initial AE1–AE2 secondary contact occurred as early as 45–52 kya in the Levant, hpEurope might have accompanied the first modern humans into Europe. However, this seems also unlikely because the presence of people from the first “Middle Paleolithic” migration Out of Africa in the Levant is not supported by archaeological evidence [55]. Thus, if initial Europeans were colonized with *H. pylori*, those bacteria were subsequently replaced by hpEurope, similar to the replacement of hspAmerind strains by hpEurope strains among Amerindians from South America [73]. To illustrate these interpretations, we show approximate routes and timings for a second colonization of Europe based on the properties of *H. pylori* populations (Figure 8), in which migration waves from North East Africa and Central Asia met and admixed in the Middle East and/or Western Asia sometimes 10–52 kya. The widespread presence of hpEurope in Mediterranean Africa is then attributed to later migrations to northern Africa, including migrations from Iberia (mtDNA haplogroup H1; 8–9 kya) [74], the Near East (mtDNA haplogroup M1; 35 kya) [75]; autosomal DNA; >12 kya [76]), or even as recently as the expansion of the Islamic caliphate in the last 1200 years. Our model also summarizes the dates of other human migrations that have distributed *H. pylori* from its southern African source (Figure 8).

### Summary

The results presented here provide a framework for the association of *H. pylori* with humans over the last 100,000 years, possibly after *H. pylori* was first acquitted by a host jump from an unknown source. This association began in Africa, where two discrete super-lineages differentiated. One of the super-lineages was predominantly associated until very recently with San (hpAfrica2) and large felines (Hac), whereas the second is widespread throughout Africa (hpAfrica1, hpNEAfrica) and accompanied anatomically modern humans during their first Out of Africa migration, which subsequently resulted in the Asian and Oceanic lineages hpAsia2 hpAsia and hpSahul. Subsequent migrations of ancestors of the African hpNEAfrica and/or the Asian hpAsia2 populations resulted in the admixed hpEurope population which then became the predominant population of extant *H. pylori* in Europe, the middle East and western Asia. We have provided date estimates for most of these historical events, thus providing a paradigm for the long-term historical reconstruction of the evolutionary path of a bacterial species.

## Materials and Methods

### Strains and ethics statement

Esophagogastroduodenoscopy was performed with written informed consent at the Interventional GI-Endoscopy Department of the Unitas Hospital in Pretoria, South Africa under ethics certificate 32/2007 (University of Pretoria, Faculty of Health Sciences Ethics Committee), with the permission of the San Council of South Africa and with permission of the ethics committee of the Charité hospital in Berlin, Germany (ethics certificate EA1/071/07). Biopsies were obtained from the antrum and corpus of the stomachs of 30 self-proclaimed San individuals. Among these were 9 Khomani San from South Africa, 11 !Xun from southern Angola, 8 Khwe from northern Namibia, and two individuals of unknown San ethnicity (Table 1, Table 2). Gastric biopsies were also taken from the stomachs of 42 anaesthetized chimpanzees (*Pan troglodytes*) undergoing annual medical examinations at the Ngamba Island Chimpanzee Sanctuary on Lake Victoria, Uganda. The examinations were conducted in full accordance with guidelines set by the International Primatological Society, Pan African Sanctuaries Alliance (PASA) and standard operating procedures by the Chimpanzee Sanctuary & Wildlife Conservation Trust (CSWCT) all of which practice the highest welfare standards for chimpanzees in captivity. Collection of biopsies was also approved by the Uganda Wildlife Authority (UWA) and ethical approval was obtained from the Uganda National Council for Science and Technology (UNCST certificate NS 71). These chimpanzees were all illegally captured in the wild as infants, but have since been confiscated and donated to the island sanctuary. All biopsies were placed in transfer medium, frozen immediately in liquid nitrogen, and kept at −80°C until transfer by courier in a liquid nitrogen dry shipper to the Max Planck Institute for Infection Biology in Berlin (CITES permit Sn. UG 001944), where we attempted to culture *Helicobacter* from them.

Gastric biopsy specimens were grown on cultivation plates containing GC agar (Remel, Lenexa, USA) supplemented with 10% (v/v) donor horse serum (Biochrom KG, Berlin, Germany), VITOX vitamin supplement (Oxoid, Basingstoke, UK) and selective antibiotics (10 mg/L vancomycin, 5 mg/L trimethoprim, 5 mg/L amphotericin B and 25000 U/L polymyxin B). The plates were incubated for 3 to 5 days at 37°C in a Forma Series II 3110 Water-Jacketed CO_2_ incubator (Thermo Scientific) wherein the CO_2_ concentration was kept at 5% and the O_2_ concentration was regulated to 5% through a mixture of H_2_ (10%) and N_2_ (90%), which facilitates efficient cultivation of microaerophilic bacteria. In case of lack of bacterial growth, the plates were incubated for up to 15 days.

We attempted to cultivate *Helicobacter* from a total of 81 chimpanzee gastric biopsies, one from the antrum and one from the corpus region of each of the 42 chimpanzees except for 3 individuals from which only one biopsy each was cultured. However, none of the chimpanzee biopsies yielded cultures of *Helicobacter*-like species. Since some of these *Helicobacter*-like species may not be detectable through bacterial culturing, a culture-free approach of 16S rRNA amplification was utilised. DNA was extracted from chimpanzee gastric biopsies with the DNeasy Blood and Tissue Kit (Qiagen). Due to the low levels of bacterial DNA that were expected, universal prokaryote 16S rRNA primers F24 (5′-GAGTTTGATYMTGGCTCAG) and F25 (5′-AAGGAGGTGWTCCARCC) were used to perform an initial PCR. This was used as a template for a second round of PCR using *Helicobacter*-genus specific 16S rRNA primers C97 (5′-GCTATGACGGGTATCC) and C05 (5′-ACTTCACCCCAGTCGCTG). These primers should yield a final amplicon of 1200 base-pairs. The PCR reaction (30 µl) contained 10× PCR Buffer (Qiagen), 330 µM dNTPs, 5 µM of each primer, 5 U of Taq polymerase and 5 µl (Biopsy) or 2 µl PCR product as template DNA. Amplification conditions were as follows: An initial denaturation at 96°C for 5 minutes followed by 35 rounds of denaturation at 96°C for 30 seconds, annealing at 58°C for 30 seconds and extension at 72°C for 90 seconds. A final extension step of 72°C for 10 minutes was then performed. After the initial enrichment PCR, a 2 µl aliquot of the PCR mixture was used as template for the second PCR. A 5 µl aliquot was examined by electrophoresis on a 1% agarose gel containing a 1/10000 dilution of Sybrsafe dye, and visualized under UV light. Using both culture dependent and culture free methods, no *Helicobacter*-like species were detected in any of the 42 chimpanzees despite intimate association between some chimpanzees and their caretakers.

Since simultaneous infection with multiple, distinct *H. pylori* strains has been frequently observed among people from Southern Africa [2], four colonies per San individual were analyzed from the stomach biopsies, two from the antrum and two from the corpus. DNA was extracted from cultures grown after single colony isolation using a DNeasy Blood and Tissue kit (Qiagen). The forward and reverse strands of fragments of *atpA*, *efp*, *mutY*, *ppa*, *trpC*, *ureI*, *yphC* were sequenced from each isolate as previously described [3], [7]. All sequences, primer combinations, PCR conditions and information on isolates are publicly available at http://pubmlst.org/helicobacter, where the new isolates described here are listed as ID numbers 1472–1527. The sequences from all seven housekeeping gene fragments were concatenated to form a 3,406 base-pair sequence. The strains cultured from the 30 San individuals (Table S1) represented 56 unique haplotypes that were used for further analysis (Table 2).

### mtDNA Analysis

DNA was extracted from San and Bantu (Northern Sotho) blood samples taken under ethics certificates 32/2007 (University of Pretoria, Faculty of Health Sciences Ethics Committee) using the DNeasy Blood and Tissue Kit (Qiagen). Assignment of individual samples to mtDNA macro-haplogroups L0–L6, M, N and R were done using a SNaPshot minisequencing procedure [77]. The mtDNA control region was amplified and sequenced following previously published methods [77], [78]. Sequence data were obtained for hypervariable segments I (HVS I; nucleotide positions 16024–16400) and HVS II (nucleotide positions 57–302), and used to assign individuals to haplogroups and sub-haplogroups according to the nomenclature proposed by Behar et al., 2008 [16].

### Population assignment of bacterial haplotypes

The 56 unique haplotypes from San were analysed together with a previously described global data set of 1040 haplotypes [7], [8] as well as 83 haplotypes from South African Bantu of Northern Sotho and Xhosa ethnicities [2], [6]. The “no admixture” model of the program Structure V2.0 [5] was used to assign individual strains to the known bacterial populations (Figure 1) [6]–[8]. New populations were not detected. Subsequent analyses were performed exclusively on African isolates, for each of the test number of populations (*K*) ranging from 2 to 5 (Figure 4). Each set of conditions was tested in ten independent Structure runs, with consistent results.

### Ancestral composition

The linkage model of Structure V2.0 [5] was used to assess the ancestral composition of individual haplotypes in order to differentiate whether populations arose as a result of gradual genetic drift or by hybridisation of two distinct populations that have come into secondary contact. We identified the previously reported populations ancestral EastAsia, ancestral Europe1 (AE1), ancestral Europe2 (AE2), ancestral Africa1, ancestral Africa2 [6], [7] and ancestral Sahul [8]. Runs assuming *K* = 4 were used to determine the ancestral composition of the European and African haplotypes displayed in the Distruct [79] plot in Figure 4B.

### Population structure of hpAfrica2

The relatedness among the strains within hpAfrica2 was analysed using the software ClonalFrame v1.1 [27]. This software estimates the clonal (vertical) genealogy of a set of DNA sequences by jointly simulating mutation and homologous (horizontal) recombination events under a neutral coalescent using a Bayesian Markov chain-Monte Carlo (MCMC) framework. Inferred horizontal events at each node are discarded for the calculation of node height, but they are used to infer common ancestry between lineages, further adding to the robustness of the genealogical reconstruction. The resulting phylogeny therefore represents the best estimate of a clonal genealogy that is currently computable. ClonalFrame phylogenies have been used successfully in resolving human demographic events in other parts of the world [8]. Bayesian parameter space was explored with 100,000 iterations, recording the posterior sample every 100 iterations, and discarding the first 10% of iterations as burn-in. This analysis was repeated 100 times, and an 80% majority rule consensus of all the sampled genealogies was computed using Treefinder [80]. Nucleotide diversity and 95% confidence limits (π_95_) within San and non-San (Bantu) hpAfrica2 strains were calculated in DnaSP4 [81], as were comparisons within San and non-San hpAfrica1 isolates.

### Multilocus data from *Helicobacter cetorum*

*H. cetorum* is a gastric *Helicobacter* from dolphins and whales and is the closest known relative of *H. pylori* according to rRNA sequences (Figure 2) [15]. It was used as an outgroup for genealogical reconstruction. In order to obtain the MLST sequences of *H, cetorum*, a draft sequence (169 contigs, 20 fold coverage, total contig length 1,744,916 bp) of the genome of *H. cetorum* strain MIT 99-5665 was obtained by shotgun sequencing with a Roche/454 Genome Sequencer FLX. *H. cetorum* sequences corresponding to the seven *H. pylori* housekeeping gene fragments were identified by BLAST searches. All sequences were confirmed by Sanger sequencing of PCR fragments amplified with the primers shown in Table S2. The sequences were submitted to the EMBL database (accession numbers: FB908911–908917).

### African populations in a global context

We used an individual as well as a population approach to determine the structure of the African *H. pylori* populations relative to other populations distributed in other parts of the world.

We again used ClonalFrame (v1.1) to estimate a clonal genealogy from multilocus sequence data of 91 globally distributed *H. pylori* strains, rooted with *H. cetorum*. This global phylogeny was estimated 100 times as above, but with each independent run recovering the same nodal topology (Figure 6A).

Recombination between closely related strains can also introduce no visible change or single nucleotide changes that resemble point mutations. These would tend to bias the ratio of rates of recombination and mutation (the rho/theta parameter) leading to an overestimation of node height. ClonalFrame corrects for this by simultaneously estimating the ratio of rates at which recombination and mutation introduce differences (the r/m parameter) which is less likely to be affected by this kind of recombinational event. To further control for the possibility of a non-linear relationship of ClonalFrame node height with time, we used the software IMa [33] for an independent, population-based estimate of the global structure within *H. pylori*. We chose Hey and Nielsen's [33] model of isolation with migration to analyze these data because it does not assume that the two populations are at equilibrium for mutation, drift or migrations. Furthermore, the model also assumes that gene flow was possible after the time of population splitting, and using a Bayesian approach, simultaneously estimates the posterior distributions of the following model parameters: time since population split (*t*), the population parameter theta (θ) and the migration parameter *m*. Sequences were first processed by the four-gamete criterion [35] implemented in DnaSP4 [81] in order to identify recombinant blocks of DNA sequence between pairs of populations. These blocks were omitted from the data set, resulting in between 42 and 100 non-recombinant blocks, depending on the pair-wise comparison, that were coded as separate loci. Treating these separate blocks as separate loci was chosen because composite likelihoods tend to estimate the true posterior probability of a parameter when the number of loci is high [82]. Bayesian parameter space was heuristically sampled by an MCMC simulation of 1,000,000 iterations, and genealogies were sampled every 100 iterations after a burn-in of 100,000 iterations. Mixing and convergence was stimulated by 100 geometrically-heated Metropolis-coupled chains, with 100 chain swapping attempts between iterations. All estimates were taken after joint parameter maximization. The analysis was repeated 10 times to determine whether MCMC simulations converged to a similar result. Among the analyses of African populations, all four pair-wise comparisons yielded consistently unimodal posterior distributions of the TMRCA, suggesting that these pairings constituted monophyletic groups. All four pairings (Table 4) were consistent with the topology of the global genealogy generated by ClonalFrame (Figure 6A), which confirmed the ancestral branching of hpAfrica2 and showed that hpNEAfrica and hpAfrica1 are sister populations. The highest and lowest values for each set of 10 simulations were regarded as the spread of the mean *t*.

### Date estimates

ClonalFrame and IMa were used to determine lineage and population coalescence respectively, both using the global rate minimum deformation (GRMD) rate-smoothing optimisation in Treefinder [80]. GRMD is a rate-smoothing method that minimises a cost function to maintain rates along different lineages that are as similar to each other as possible, within the imposed time boundaries. This method is appropriate given the linear relationships of ClonalFrame's node height and IMa's *t* with calibration time (Figure 5). The spread of node heights of the 100 ClonalFrame genealogies and the spread of *t* was combined with six known calibration points (Table 3), where node height and *t* values had been previously determined [8], to generate TMRCA estimates. We used a Treefinder [80] script (Text S1) to generate 95% confidence limits from the spread in *t* values. The ranges of population and individual-based TMRCA dates were found to overlap for all but one case, but the confidence limits returned by the IMa were greater. The upper and lower values described in Figure 6 and in the text are the highest and lowest values that were estimated using both methods.

### Matrix regression

Pair-wise population divergence estimates were obtained in Mega [83], using maximum composite likelihood distances for both concatenated *H. pylori* sequences (n = 485, Table S3) and whole genome (or coding region) human mtDNA sequences (n = 447, Table S4). A Mantel test was used to perform a distance matrix regression in GenAlEx [84]. The probability that a random regression co-efficient was greater than or equal to the observed value was determined by 9999 permutations.

### 16SrRNA Phylogeny

We obtained 16SrRNA sequences of the following various gastric and enterohepatic Helicobacter species from Genbank and/or extracted the 16SrRNA sequences from complete genomes: *H. pylori* (accession number AE000511, human host), *H. acinonychis* (AM20522, lion), *H. cetorum* (AY143177, dolphin), *H. felis* (M37643, cat), *H. bizzozeronii* (Y09404, dog), *H. salomonis* (U89351, dog), *H. cynogastricus* (NR_043457, dog), *H. heilmannii* (HM625820, cat), *H. baculiformis* (EF070342, cat), *H. suis* (AF127028, pig), *H. bovis* (AF127027, cattle), *H. macacae* (HQ845265, rhesus monkey), *H. canadensis* (AF262037, human), *H. cholecystus* (U45129, hamster), *H. cinaedi* (M88150, human), *H. bilis* (U18766, mouse), *H. canis* (L13464, dog), *H. hepaticus* (AE017125, mouse), *H. muridarum* (M80205, rodent), *H. pullorum* (L36144, chicken), *H. trogontum* (U65103, rat), *H. fennelliae* (M88154, human), *H. rodentium* (U96296, mouse), *H. mesocricetorum* (AF072334, hamster) plus *Wolinella succhinogenes* (M88159, cow) and *Campylobacter jejuni* (AL111168, chicken) as outgroups. The aligned and trimmed sequences were used to generate a Neighbor-joining tree (Figure 2) using the Maximum Composite Likelihood algorithm in Mega [85].

### Accession numbers

http://pubMLST.org/helicobacter isolate ids:1472–1527.

## Supporting Information

Table S1 Mitochondrial DNA haplotypes, number of *H. pylori* cultures and unique *H. pylori* haplotypes per individual.
(XLS)

Table S2 Primers designed from a whole genome alignment and used to amplify and sequence the 7 homologous housekeeping gene (MLST) fragments in *Helicobacter cetorum*.
(XLS)

Table S3 *H. pylori* sequences used in Mantel regressions.
(XLS)

Table S4 Source of human mitochondrial DNA sequences used in Mantel regressions.
(XLS)

Text S1 Treefinder script to generate confidence limits from the spread of posterior IMa *t* values.
(TXT)
